# Effect of mannan oligosaccharides on the microbiota and productivity parameters of *Litopenaeus vannamei* shrimp under intensive cultivation in Ecuador

**DOI:** 10.1038/s41598-020-59587-y

**Published:** 2020-02-17

**Authors:** Oreste Gainza, Jaime Romero

**Affiliations:** 1 Departamento de Acuicultura, Universidad Católica del Norte, Doctorado en Acuicultura, Programa Cooperativo Universidad de Chile, Pontificia Universidad Católica de Valparaíso, Universidad Católica del Norte, Larrondo 1281, Coquimbo, Chile; 2 Laboratorio de Biotecnología de Alimentos, Unidad de Alimentos, Instituto de Nutrición y Tecnología de los Alimentos (INTA), Universidad de Chile, El Líbano 5524, Macul, Santiago, Chile

**Keywords:** Microbiome, Biological sciences

## Abstract

The white leg *Litopenaeus vannamei* shrimp is of importance to the eastern Pacific fisheries and aquaculture industry but suffer from diseases such as the recently emerged early mortality syndrome. Many bacterial pathogens have been identified but the *L*. *vannamei* microbiota is still poorly known. Using a next-generation sequencing (NGS) approach, this work evaluated the impact of the inclusion in the diet of mannan oligosaccharide, (MOS, 0.5% w/w), over the *L*. *vannamei microbiota* and production behavior of *L*. *vannamei* under intensive cultivation in Ecuador. The MOS supplementation lasted for 60 days, after which the shrimp in the ponds were harvested, and the production data were collected. MOS improved productivity outcomes by increasing shrimp survival by 30%. NGS revealed quantitative differences in the shrimp microbiota between MOS and control conditions. In the treatment with inclusion of dietary MOS, the predominant phylum was Actinobacteria (28%); while the control group was dominated by the phylum Proteobacteria (30%). MOS has also been linked to an increased prevalence of *Lactococcus-* and Verrucomicrobiaceae-like bacteria. Furthermore, under the treatment of MOS, the prevalence of potential opportunistic pathogens, like *Vibrio*, *Aeromonas*, *Bergeyella* and *Shewanella*, was negligible. This may be attributable to MOS blocking the adhesion of pathogens to the surfaces of the host tissues. Together, these findings point to the fact that the performance (survival) improvements of the dietary MOS may be linked to the impact on the microbiota, since bacterial lines with pathogenic potential towards shrimps were excluded in the gut.

## Introduction

The “white leg” *Litopenaeus vannamei* shrimp is originally from waters of the equatorial Pacific east coast. Commercial cultivation began in Ecuador in 1968 in the province El Oro where this study was performed^1^. The shrimp industry has also been significant in other regions, such as the Middle East, Southeast Asia, the Indian Subcontinent and China. The production of the shrimp industry in Latin American countries such as Brazil, Honduras, Mexico and Ecuador, is relevant within the aquaculture economic context in the region^2^. Despite this apparent success in terms of production expansion, global shrimp production continues to suffer significant losses due to the effects of a wide range of diseases^3^. In recent years, Acute Hepatopancreatic Necrosis Syndrome (AHPNS), has impacted *L*. *vannamei* production areas worldwide, leading to major economic losses and significantly affecting production^4^. This disease is caused by *Vibrio parahaemolyticus*, which colonizes the digestive tract and releases toxins that affect the liver and pancreas^5^.

Traditionally, fight diseases strategies in aquatic cultures have been based on the use of antibiotics and chemotherapeutics; currently, however, the biosafety directives of markets and the environmental legislation of producing countries have limited this approach. The employment of antibiotics for the control of the mass outbreaks of bacterial diseases in the cultivation of shrimp has raised the possibility of the emergence of bacterial strains resistant to antibiotics due to the selective pressure exerted by antibiotic residues in environment (e.g., bottoms, sediments) and by the unintentional exposure of cultivated or wild shrimp to antibiotics^6–9^. Currently, it is widely accepted that prevention is more advisable than treatment; thus, strategies have been developed to modify the intestinal microbiota with the goals of promoting colonization of beneficial bacteria and of preventing the colonization of potentially pathogenic bacteria^9,10^.

Considering the intestinal microbiota as the microbial ecosystem colonizing the digestive tract, which serves as the primary interaction surface between the external environment and the internal environment of any organism, numerous investigations in humans and other vertebrates have led to the comprehension of the essential importance of microbiota to health and welfare. In fish cultivation, evidence has been found regarding the contributions of microbiota to various aspects of the production of enzymes to improve nutrient availability and the competitive exclusion of potential pathogenic bacteria^10–16^. Other studies have provided evidence about the of microbiota´s function in host nutrition, specific tissues proliferation and immune mechanism regulation^17–20^. For example, bacterial strains derived from intestinal microbiota such as *Pediococcus acidilactici*^21–23^ and *Bacillus subtilis*^24–26^ have been tested in various aquaculture species and shown beneficial effects Thes.e advances have fostered a growing interest in microbiota modulation and their consequences on the productivity performance of animals under cultivation^9,10,27^. These supplements may be (i) probiotics, defined by Merrifield *et al*.^28^ as any microorganism supplied through the feed or culture water that benefits the host; (ii) prebiotics, defined by Bindels *et al*.^29^ as an indigestible compound which, via its metabolic breakdown by microorganisms in the intestine, acts on the profile and functionality of the intestinal microbiota, giving it a positive action on the host as an improvement of food growth and efficiency; or iii) symbiotic, which are defined by Gibson & Roberfroid (1995)^30^ as nutritional supplements containing a prebiotic and a probiotic that beneficially influence the host by acting on the survival and implantation of live microorganisms as dietary supplements in the gastrointestinal tract.

Mannan Oligosaccharides (MOS) are glucides obtained from the *Saccharomyces cerevisiae* yeast cell^31,32^. The use of MOS to block pathogen colonization derives from the conception that certain polysaccharides could be used to block the mechanism of recognition and adhesion of potential pathogens to molecules on the surfaces of host tissues (competition for attachment sites). This action would reduce the adhesion of the pathogens to the digestive tract, leaving them to be excreted in the feces. This may lead to the improvement of the integrity and performance of the intestinal epithelial barrier^16^.

There are no previous publications using deep sequencing (next generation sequencing, NGS) to address effects of MOS in *L*. *vannamei* microbiota. Several investigations have reported the effects of MOS on crustacean culture performance including parameters such as growth rates, survival, hemocyte proliferation, polyphenoloxidase activity, and changes in gastrointestinal (GI) morphology. In terms of microbiological analyses, only quantitative variation of aerobic bacteria has been described^33–43^. However, these previous investigations were conducted under laboratory conditions and involved only cultivable bacteria. Therefore, these observations have not been validated at a commercial farming scale, limiting the application of those prebiotics.

For cultivated crustaceans such as *L*. *vannamei*, information on the functionality and structuring of microbiota is very limited. Latterly, advances in NGS have allowed develop research on crustaceans microbiota, as recently addressed in some studies^44–48^. These investigations have been based on modulating the *L*. *vannamei* microbiota through nutritional challenges in laboratory conditions. However, information is lacking regarding the microbiota-host relationship, including evidence of cause and effect relationships on the physiology of the host and consequences for productivity. Considering this deficiency, our research was aimed to use NGS to assess the effects of the dietary inclusión of a prebiotic (MOS) on both: production parameters in intensive cultivation conditions and composition of the intestinal microbiobiota of *L*. *vannamei* in Ecuador.

## Results

### Productivity response indexes to the inclusion of MOS

**Figure 1:**
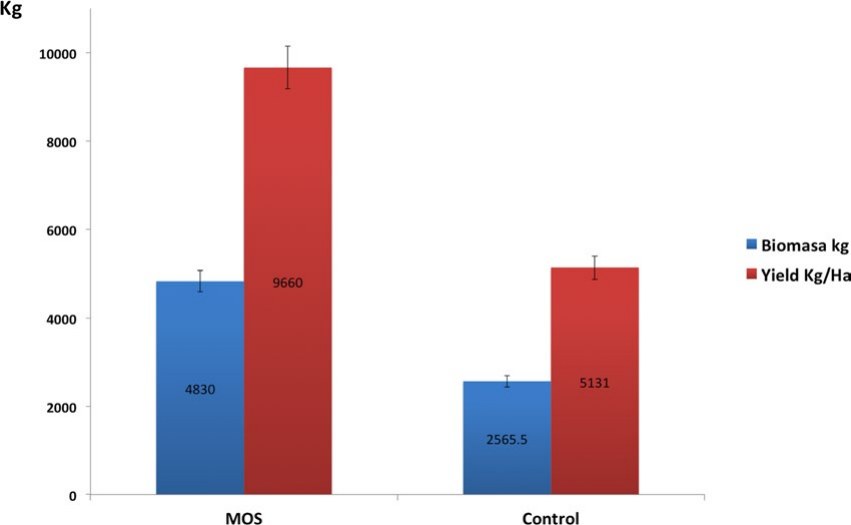
Productivity outcomes. Graphic representation of the average harvested biomass (Kg) and yield (Kg/Ha) of *L*. *vannamei* in ponds supplemented with mannan oligosaccharides (MOS) and ponds with a commercial diet (Control).

The shrimp population in all the ponds (MOS and control) was completely removed because the entire biomass was harvested for the market. This action was coordinated with the new moon phase because this was the period during which the largest proportion of the shrimp was in Stage C (anecdysis) of the molting cycle^49^. The influence of the lunar cycle on *L*. *vannamei* commercial culture, has long been recognized in Ecuador from the observation of molting patterns and activity cycles in aquaculture production ponds, and its follow-up is financially relevant due to the fact that soft shrimp cause high post-harvest handling losses^50^. Therefore, because ecdysis corresponds to the renewal of the exoskeleton during the molting cycle, for practical reasons, the harvest should be performed during anecdysis^51^. Cultivation outcomes (Fig. 1) revealed better performance in ponds whose populations received feed supplemented with MOS, based on the total harvested biomass (B) and the performance by cultivation area (R). The comparative analysis in Table 1 reflects significant increases in B and R associated with the treatment with 0.5% MOS and highlights a 34% increase in survival (S). Meanwhile, the productivity response, expressed in terms of average weight (W), food conversion (CV) and weight gain (WG), exhibited no significant dissimilarities (p > 0.05).

**Table 1:** Indexes used for evaluation of the productivity outcome. (W; CV; WG; B; R), Welch’s t-test^74^. (S), Contingency tables using Pearson’s *X*^2^ test^75^. Source of data: Industrial biomass payment report.

Productive Performance Indicators	0.5% MOS	Control	P value
Average weight W (g)	15.6 ± 0.6	15.5 ± 0.7	0.978
Food Conversion CV	1.2 ± 0.1	1.63 ± 0.02	0.081
Weight Gain WG (g/d)	0.2 ± 0.0	0.2 ± 0.0	0.861
Average Biomass B (kg)	4830 ± 189	2565 ± 201	0.028*
Yield R (kg/Ha)	9660 ± 378	5131 ± 402	0.028*
Survival Rate S (%)	70.3 ± 6.6	37.5 ± 4.7	2.2E-16**

### 16S rRNA gene sequencing

**Figure 2:**
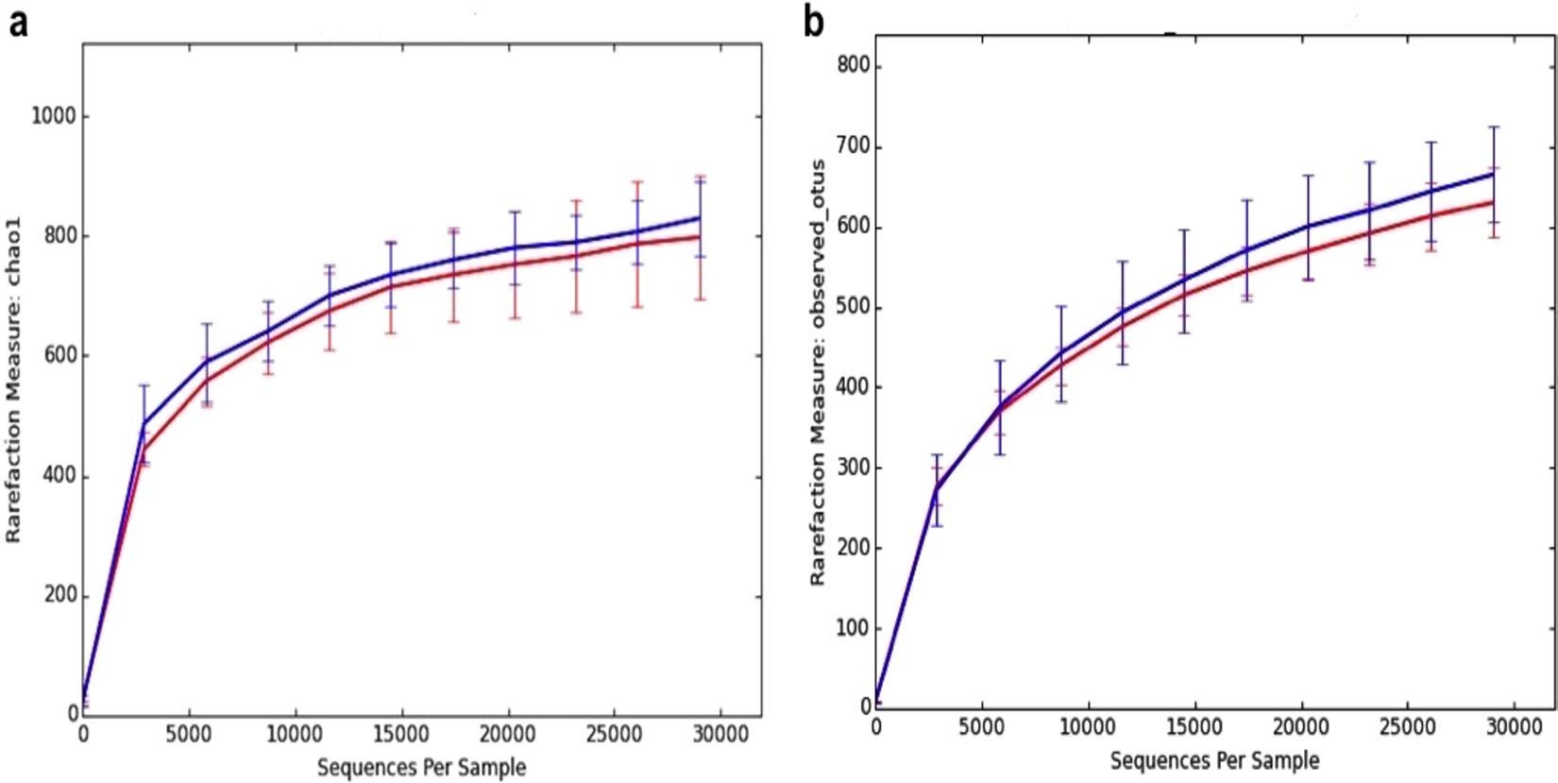
Rarefaction curves. (**a**) Alpha diversity index Chao1: Species richness estimators based upon the number of rare OTUs found in a sample. (**b**) OTUs observed: Richness is expressed as the number of observed OTUs. Curves were averaged by ■ Control, ■ 0.5% MOS for a sampling depth of 29,000 sequences. Errors bars represent standard error.

Sequencing of the 16S amplicons with Ion Torrent technology provided a total of 1,303,492 sequences, having an average-quality ratio by sequence (Phred Score) of 29, corresponding to a base call accuracy of 99.9% (Supplementary Table 1) plus a probability of error of 0.00126. The data of the sequences were deposited in the Sequence Read Archive of the National Center for Biotechnological Information (SRA, NCBI) in the framework of the BioProject (PRJNA352369). After dereplication and removal of chimeras, singletons and Archaea sequences, were a global of 470,639 sequences of high quality (Table 2), that were classified to a total of 2065 operational taxonomic units (OTUs), 97% of which were classified by sequence identity. Bacteria were the focus of this study, and archaeal sequences were detected unintentionally due to mismatches in the primers used. The rarefaction curves for OTUs observed and the Chao1 alpha diversity index approached the saturation phase, indicating good sample coverage, while the asymptotic distribution of the curves implies that they are comparable (Fig. 2).

**Table 2:** Numbers of sequences per sample.

Pond	Condition	Sequences/sample
P2	MOS	40,814
P2	MOS	42,611
P2	MOS	46,966
P6	MOS	30,625
P6	MOS	36,558
P6	MOS	42,725
P10	Control	56,553
P10	Control	70,436
P10	Control	103,351
	**Total**	470,639

### Alpha diversity

The alpha diversity of the microbiota was slightly higher in shrimps that were fed with the control diet compared to shrimps that were fed with the MOS diet 0.5% (Fig. 2). However, this difference was not significant (Table 3).

**Table 3:** Comparison of the alpha diversity in the microbiota of *L*. *vannamei* supplemented with MOS (nonparametric t-test) for a sampling depth of 29,000 sequences.

Process	Control mean	MOS mean	t stat	P-value
Chao1	830	795	0.491	0.639
OTUs Observed	671	636	0.866	0.398

### Beta diversity

**Figure 3:**
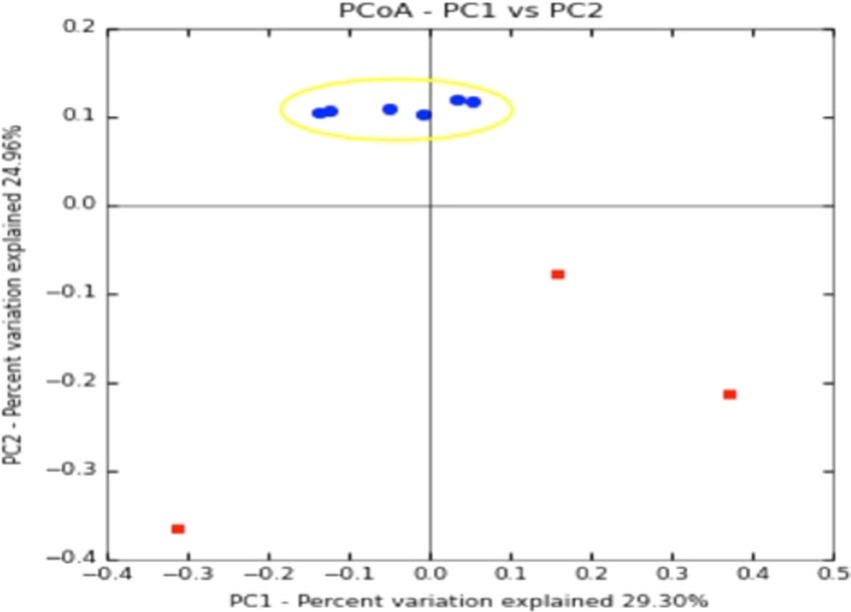
Principal component analysis (PCoA) normalized distribution plot. Blue dots correspond to microbiota of shrimp supplemented with MOS, and red dots correspond to microbiota of shrimp fed the commercial diet as described in Material and Methods.

The non-directional variation of the beta diversity in a community is a measurement of the change of the community structure as a response to environmental or experimental factors^52^. Beta diversity linked to *L*. *vannamei* bacterial communities in the two conditions, control and 0.5% MOS dietary supplementation was investigated through PCoA (Fig. 3). The first 2 categories cover a total of 54.26% of the variation (first component, 29.3%; second component, 24.9%). The results of the similarity analysis test (ANOSIM), with R = 0.6 ≈ 1 (p = 0.016), showed that there are significant variations among the microbiota of the animals treated with MOS and those of the control animals. The multivariate analysis of the taxa that support this diversity is described in the following sections.

### Comparison of the intestinal microbiota

**Figure 4:**
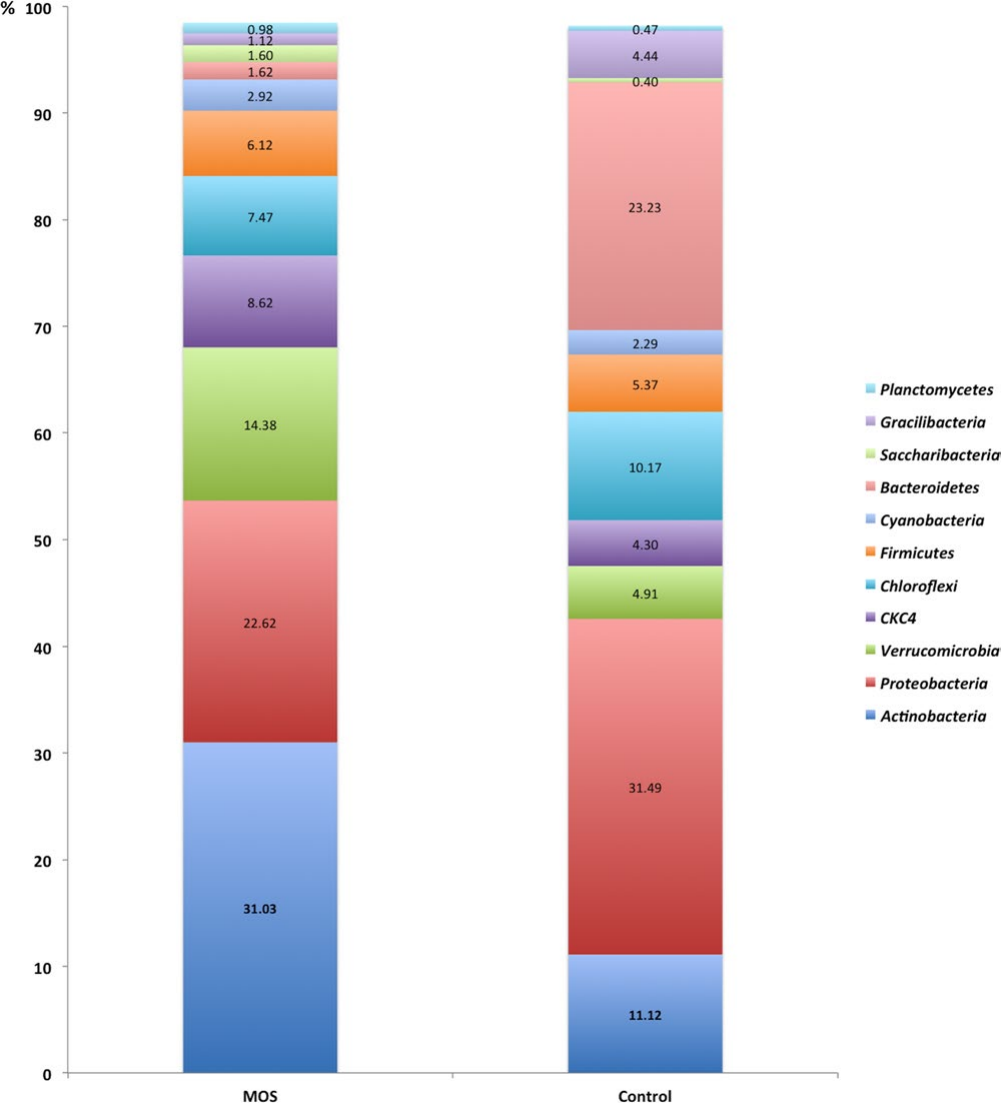
Comparison of relative abundance at the phylum level. Shrimp microbiota composition (relative to OTU composition) at the phylum level. Comparison between Control and MOS shrimp microbiota including the 11 phyla showing the highest abundance. Taxonomic summary of the observed relative abundance of abundant phyla across all samples divided by culture condition.

The prevailing sequences linked to MOS treatment at the Phylum level were mostly Actinobacteria (28%), Proteobacteria (20%), Verrucomicrobia (13%), Chloroflexi (7%) and Firmicutes (6%), whereas the microbiota in the control group were dominated by Proteobacteria (30%), Bacterioidetes (22%), Actinobacteria (11%), Chloroflexi (10%) and Firmicutes (5%) (Fig. 4). In total, 39 phyla were detected.

**Figure 5:**
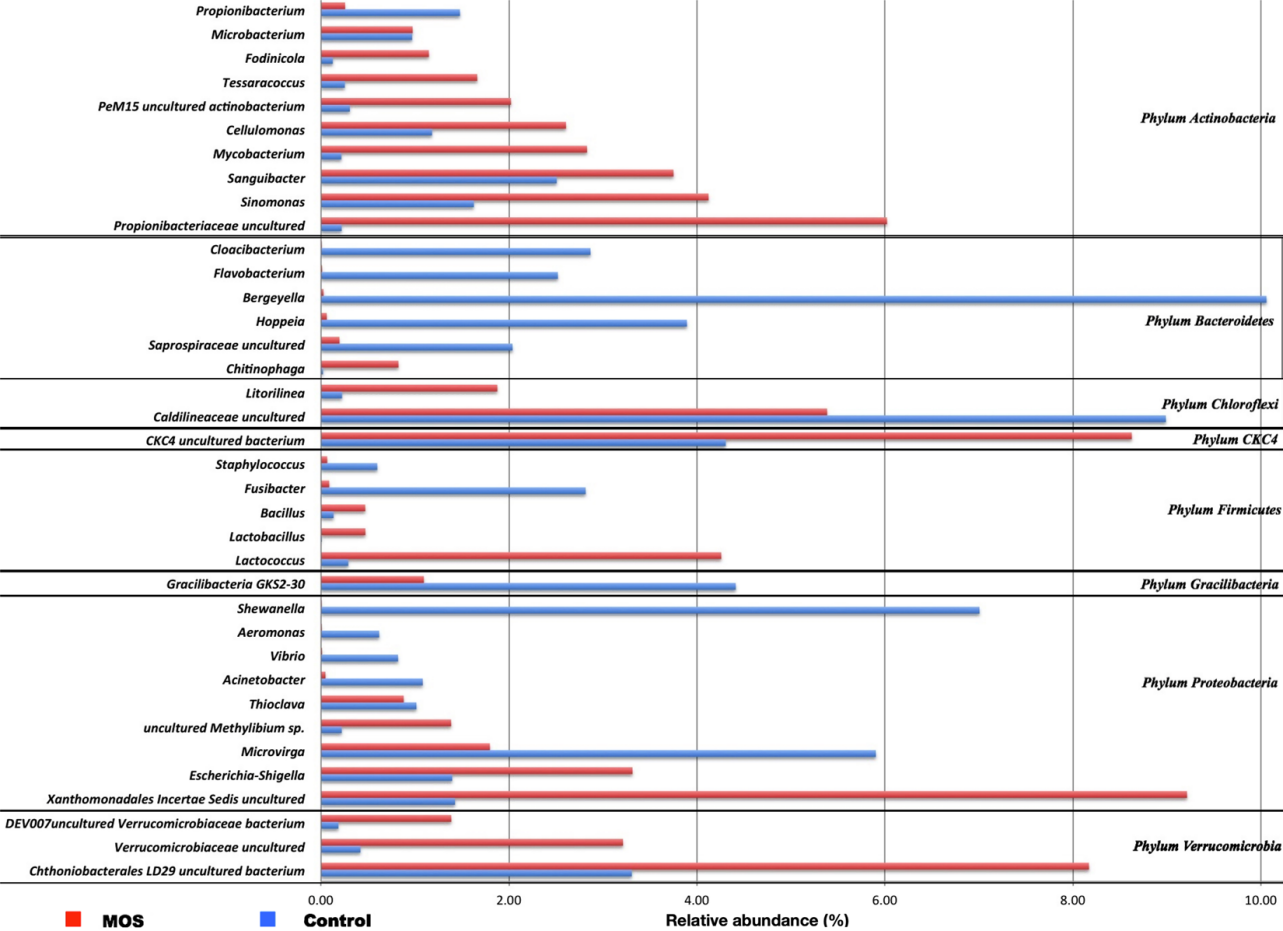
Comparison of the relative abundance of the most abundant genera between Control and MOS shrimp microbiota. Microbiota composition (relative to OTUs composition) at genus level, between Control and MOS supplemented microbiota. Taxonomic summary of observed relative abundance of abundant genera across all samples divided by culture condition.

Identification by taxonomic analysis allocated a total of 847 genera. The most abundant genera under MOS treatment were uncultured Xanthomonadales (9%), uncultured CKC4 (9%), uncultured Chthoniobacterales LD29 (8%), uncultured Propionibacteriaceae (6%) and uncultured Caldilineaceae (5%). In contrast, the control shrimp microbiota was composed by *Bergeyella* (10%), uncultured Caldilineaceae (9%), *Shewanella* (7%) and *Microvirga* (6%) (Fig. 5).

### Identifying OTUs that differed significantly between treatments

The analysis of the differential abundance considered only those OTUs with more than 100 sequences. This analysis revealed a total of 4 OTUs that were present at significantly higher levels in the MOS treatment than in the Control (p < 0.05), as follows: OTUs 62 and 43, uncultured Verrucomicrobiaceae; OTU 14, *Lactococcus;* and OTU 86, *Chlamydia*. OTUs with significantly lower presence in the microbiota of shrimp fed MOS than in control shrimp were as follows: OTU 7, *Shewanella* sp.; and OTU 1, *Bergeyella* (Table 4).

**Table 4:** OTUs with differential abundance levels observed in the analysis of differential abundance based on MetagenomeSeq’s fitZIG algorithm^86^ for OTUs with more than 100 counts.

OTU	MOS 0.5%	Control	FisherP	FisherAdjP	AdjPvalues	Taxonomy
OTU_14	6,945	380	1	1	0.046	*Lactococcus*
OTU_43	1,990	12	0.083	1	0.046	*Uncultured* Verrucomicrobiaceae
OTU_62	1214	4	0.083	1	0.046	*Uncultured* Verrucomicrobiaceae
OTU_86	611	3	0.083	1	0.046	*Chlamydia psittaci 6BC*
OTU_1	28	23,154	1	1	0.046	*Uncultured Bergeyella*
OTU_7	9	15,376	0.5	1	0.046	*Shewanella sp*. *MOLA 59*

### Linear discriminant analysis (LDA) effect size (LEfSe)

**Figure 6:**
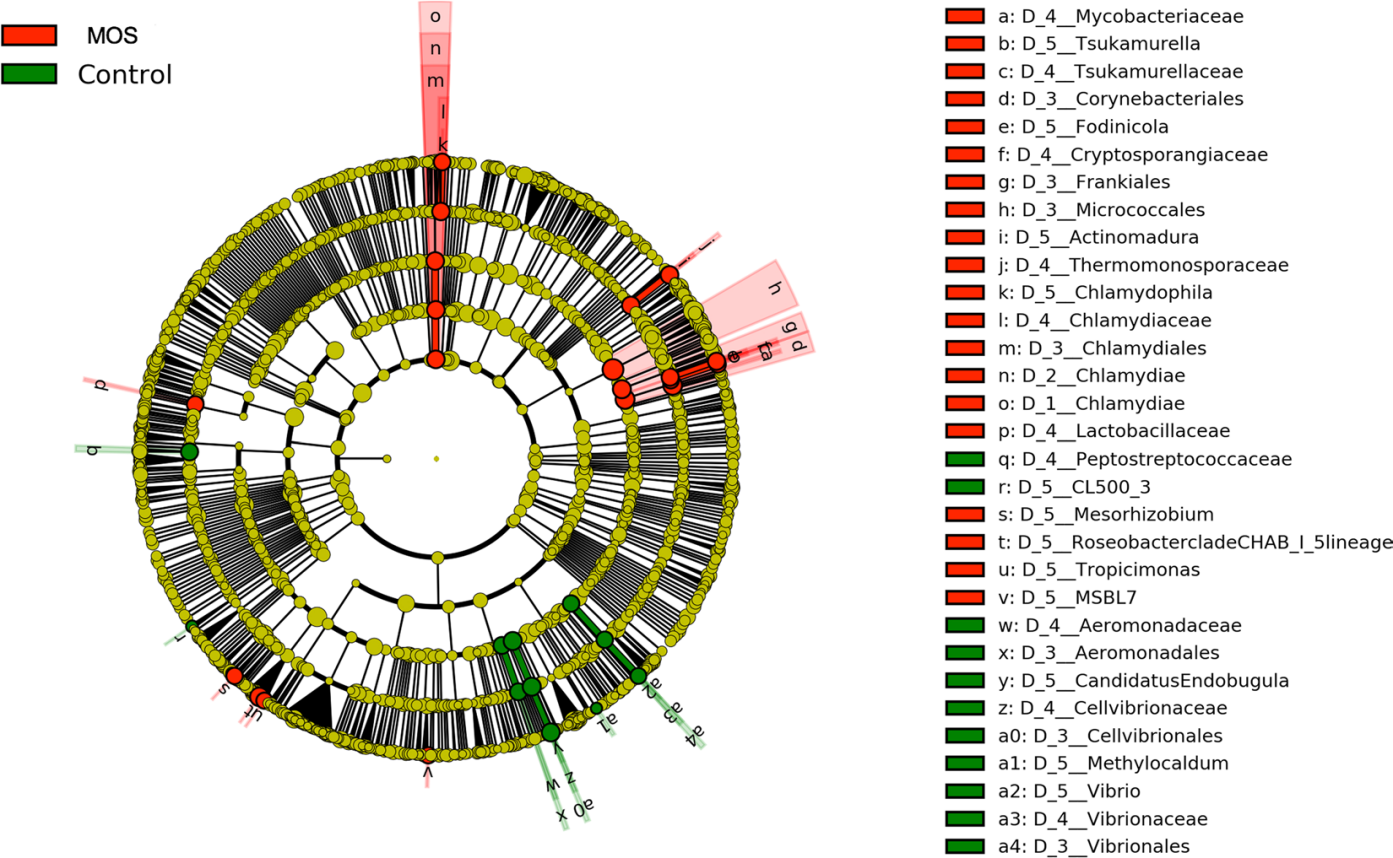
Circular cladogram information of results from LEfSe, reporting OTUs identified with significant differences p < 0.05; LDA > 2.5. Cladogram illustrating the phylogenetic relationship amongst the significantly differentiating bacterial taxa. The dots in the center present the OTUs at phylum level, whereas the outer circle of dots present the OTUs at genus level. The colors of the dots indicate the treatment (MOS or Control diet) in which the respective OTUs are most abundant. The explanation of the colors is given in the upper left corner. Yellow color indicates OTUs that showed similar abundance in both treatments. Phyla, classes, orders, families and genera that were significantly different between treatments are named along the right side of the figure.

**Figure 7:**
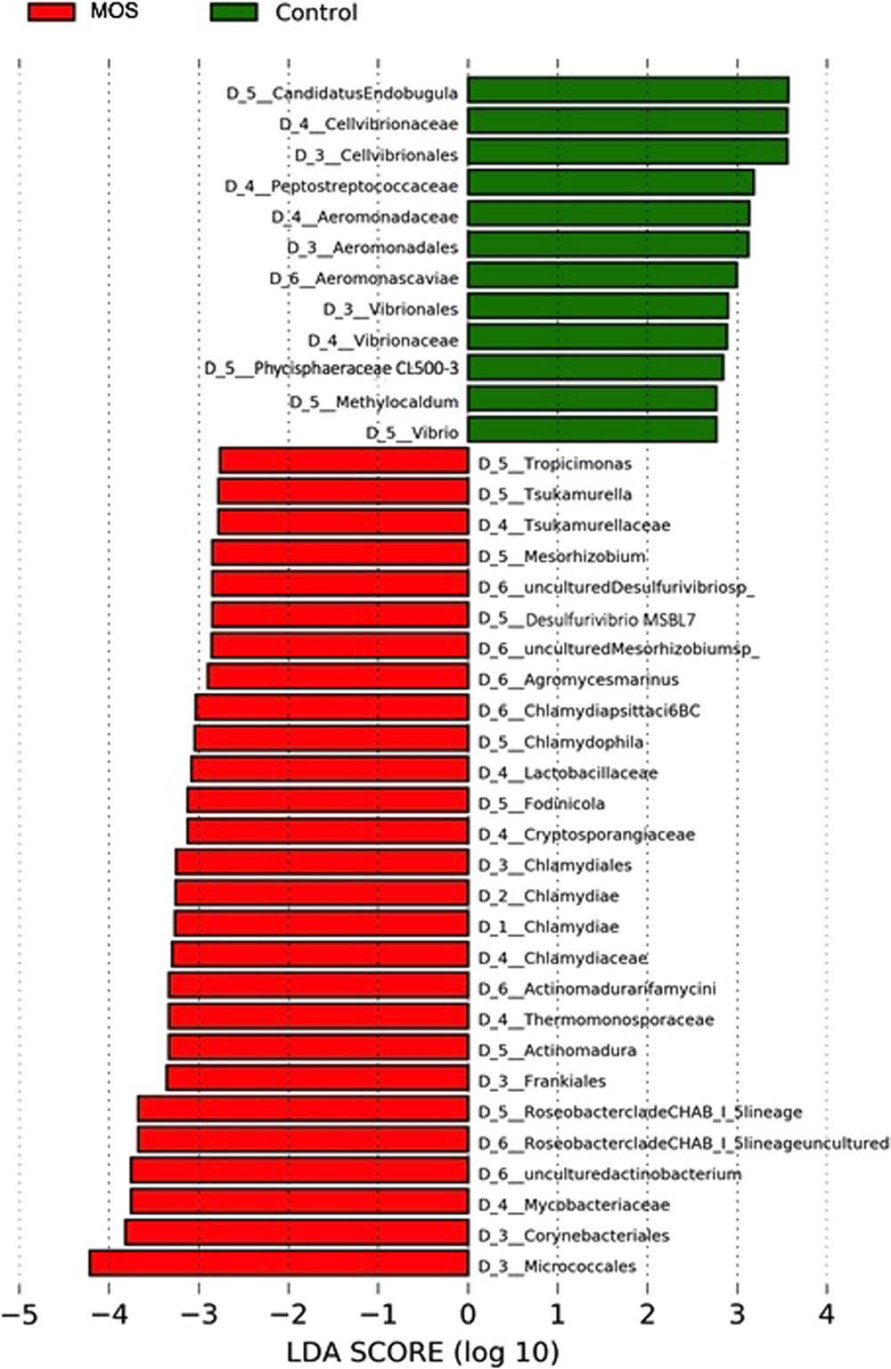
Graphical summary of LEfSe results. Comparison of LDA effect size of the significantly differentiating bacterial taxa. The histogram shows the LDA scores computed for significantly abundant taxa between Control and MOS shrimp microbiota. The histogram represents the most significantly abundant taxa between MOS and Control shrimp microbiota. The explanation of the colors is given in the upper left corner.

Using LEfSe, microbiota were compared between the Control and MOS treatments in order to identify taxa consistently associated with each condition (MOS or Control) that consistently explain their differences based on the effect size. The results of this analysis are shown in Figs. 6 and 7 and reflect the fact that MOS treatment significantly promoted 27 taxa (LDA > 2.5). Among these groups are highlighted taxa for the phylum Actinobacteria, including the orders Micrococcales, Corynebacteriales and Frankiales and the genus *Actinomadura*. In contrast, only 12 taxa were associated with the control group, and these taxa mainly belonged to the phylum *Proteobacteria*. The taxa associated with potential crustacean pathogens, such as the order Aeromonadales, the family Aeromonadaceae, the species *Aeromonas caviae* and the order Vibrionales, family Vibrionaceae and genus *Vibrio*, stood out distinctly.

## Discussion

To the best of our knowledge, this article presents the first description of *L*. *vannamei* microbiota´s diversity and taxa composition, under MOS dietary treatment in a commercial aquaculture facility. There are no previous NGS-based reports addressing the effect of MOS on the microbiota of *L*. *vannamei*. Productivity parameters have been reported previously, but those reports have been limited to laboratory conditions at the experimental level. In one study with 1080 shrimp distributed in 36 1-m^3^ tanks, Zhang *et al*.^40^ reported a 66% increase in weight gain (WG; P < 0.05) in the group treated with 0.2% MOS. In another study with 270 shrimp in 18 0.128-m^3^ aquariums, Genc & Ebeoglu 2013^41^ reported a 17% increase in survival (S) after increasing MOS to 0.4%, but these results relied on the salinity of the environment (38‰). Our results under intensive commercial cultivation conditions, with 1,780,000 juvenile *L*. *vannamei in* 4 0.5-ha ponds, 5‰ salinity and 0.5% MOS, contrast with these laboratory conditions because, unlike the report by Zhang *et al*.^40^, we found no significant differences in growth parameters between the animals supplemented with MOS and the control treatment. Our increase in harvested biomass (B) in the pond that received the 0.5% MOS treatment was a response to the significant increase in S (>30%). The observed effect matches results from S Genc & Ebeoglu, 2013^41^, but our results obtained at 5‰ contrasted with those in the previous study, which reported a positive response only to salinities greater than 38‰.

The promotion of changes in the composition of the intestinal microbiota is a key component of the case for using MOS as prebiotics, but this aspect has also been one of the least developed. The composition of the microbiota of shrimp under control conditions is consistent with previous research on this species, agreeing that Proteobacteria is the dominant phylum, with relative abundance levels between 28 and 70%^44–48,53–57^. Other phyla showing high abundance under control conditions were Bacteroidetes (22%) and Actinobacteria (11%), whose values matched those in some of the previous studies; however, overall, those studies showed variable relative abundance levels.

The effect of the inclusion of MOS on the microbiota of shrimp is reflected in the relative abundance at the phylum level, with the predominance of Actinobacteria over Proteobacteria and without any previous background about the displacement of the phylum Proteobacteria by the phylum Actinobacteria in association with a dietary intervention in this species. However, using NGS, it has been reported for *L*. *vannamei* that dietary supplementation with the supernatant from the bacterial strain HC-2^46^ or with cornstarch as a source of carbohydrates^45^ leads to changes associated with increases in Actinobacteria and Firmicutes, respectively, without affecting the position of Proteobacteria as the dominant phylum.

Previous studies of the effect of the inclusion of MOS on microbiota are based on measurements of cultivable bacteria counts, and most of those studies are focused on fish. There is no published work on the modulation of microbiota in *L*. *vannamei* by dietary MOS. However, reports in other crustaceans have described reductions in the counts of *Vibrio*^36,37,42^. This reduction in colonization of the digestive tract by potential pathogens would lead to an improvement in the integrity and functionality of the epithelial intestinal barrier^16^. Consequently, the absence of potential pathogens in MOS-treated shrimp promoted the presence of uncultured Verrucomicrobiaceae and *Lactococcus*. Competition between *Aeromonas* and *Lactococcus* for binding sites has been previously demonstrated *in vitro*^58^. Furthermore, genomes of *Lactococcus* spp. have revealed the presence of adhesins associated with their permanence in the gut^59^. Considering the impact of AHPNS on the production of *L*. *vannamei* worldwide^4^, our results showing the reduction of the genus *Vibrio* to negligible levels in a commercial culture facility are of particular relevance; previous studies only involved laboratory-scale assays^36,37,42^. This work allows us to infer that MOS could constitute an important tool for preventing AHPNS, which remains one of the most interesting open fields for exploration based on our results.

The change in the intestinal microbiota is reflected in the improved survival and better productivity outcomes and is consistent with the concept that Actinobacteria produce beneficial secondary metabolites for the host, such as antimicrobial factors and growth promoters^4,60,61^. There are numerous publications on the potential use of Actinobacteria as probiotics^4,62–65^, but they have all focused on the genera *Streptomyces* and *Lactococcus*. Meanwhile, Xiong *et al*., 2016^54^ suggest that the selection of probiotics should favor the specific microbiota of each species to provide a greater probability of intestinal colonization. Given the results obtained here, potential probiotics belonging to the genera *Actinomadura*, *Fodinicola* and *Agromyces* should be evaluated for their abundance and association with MOS (Fig. 7). On the other hand, it has been reported that the presence of *Verrucomicrobiaceae* is related to the recovery of the functionality of the intestinal epithelial barrier, since these bacteria use complex oligosaccharides as substrates for fermentation^66^. The limitation of this group may be that isolates are difficult to obtain.

The primers used in the study (341F, 518R) correspond to conserved regions that have been evaluated *in silico* for Illumina and Ion Torrent sequencing^67^. This study revealed coverage of 96.7% in Bacteria, 44.6% in Archaea and 0.2% in Eucarya while not detecting the candidate divisions OP11 and WS6 or Armatimonadetes. Those undetected phyla have not been mentioned in any previous report about the intestinal microbiota of the *L*. *vannamei*. Therefore, we consider that with the filtering of the sequences assigned to Archaea and Eucarya, we obtained a widely representative library. However, the detection of Archaea may open a new field of exploration in the description of shrimp microbiota. The abundance of Archaea (10%) may imply that these organisms play important roles within the microbiota of shrimp. For example, one of the detected archaeal taxa corresponded to Thaumarchaeota, which includes microorganisms involved in the nitrogen cycle, a critical parameter for shrimp health^68^. However, this observation must be verified using the proper primers to describe this domain.

In 2010, Daniels *et al*.^36^ stated that the action of MOS stabilizes the composition of the microbiota and partly suppresses the variations and influxes of new bacterial strains from the environment. This statement is fully consistent with the results of our diversity analysis and may be extended to say that the action of the MOS also controls the influx of bacterial strains with potential pathogenicity for *L*. *vannamei*. The key innovation of this research can be summarized as the first approach through NGS to the management of the *L*. *vannamei*’*s* microbiota modification under commercial cultivation conditions.

## Materials and Methods

### Experimental animals and environmental conditions

The study was performed at the Santa Ana intensive shrimp farm, located at 3° 31′ 10.7′′S, 80°10′ 29.74′′W, belonging to canton Huaquillas, El Oro Province, Ecuador. Santa Ana is located in the dry thorn scrub zone of the province of El Oro, between Arenillas and Huaquillas, in the altitudinal range 0–50 meters above sea level^69^. Approximately 1,780,000 juvenile *L*. *vannamei* with an average initial weight of 2.2 ± 0.53 g (calculated by routine weekly population sampling in culture procedures, n = 4000) from a breeding pond at the shrimp farm were distributed in 4 pools of 0.5 ha each with the temperatures controlled between 32 °C and 34 °C, salinity at 5‰_,_ oxygen saturation above 60%, over 60 days of cultivation. To minimize the factors that could influence the results of the experiment, we standardized conditions such as maturation stage and the conditions of the ponds in order to avoid interfering with the culture population, since a significantly representative periodic monitoring of the microbiota of culture population would require intervention in the biomass with the periodic slaughter of a large number of individuals cultivated for market. The ponds are only linked hydrologically by a common water source, with independent inputs. The ponds were constructed under greenhouse conditions, including the use of a geotextile liner for impermeabilization. Environmental variables have been parameterized throughout the culture production tracking protocol in intensive culture systems. Under commercial production conditions, there is less control of environmental variables than under laboratory conditions or experimental production, so the management protocol seeks to standardize the conditions in the production pools. We used the most common commercial feed formula, whose standardized proximal composition has been published by the manufacturer (Nicovita Classic Camarón Vitapro, Callao, Peru; Bio-Mos®, Alltech Inc., Nicholasville, KY, US).

### Food and treatment

The shrimps were fed 4 times per day (08:00, 11:00, 14:00 and 17:00), cultured for 59 days, until they reached their harvest average weight (total body weight W ± 14 g; calculated by routine weekly sampling, n = 1000), with feed adjustments according to demand at the trough. A 2.0 mm commercial feed from Nicovita Classic Camarón (Vitapro, Callao, Peru) was used; the composition reported by the manufacturer was 35% protein, 5% fat, 10% ash, fiber 5% and 12% moisture. The inclusion of 0.5% (w/w) of MOS (Bio-Mos®, Alltech Inc., Nicholasville, KY, US) to the feed was through the dilution of MOS in distilled water (0.125 g/mL) plus commercial gelatin (0.125 g/mL), sprayed onto the feed (0.04 mL/g) in the mechanical hopper for homogenization. The food for the two control pools was prepared with the same protocol with the addition of commercial gelatin without MOS.

### Sampling procedure

Complete harvesting is usually undertaken by using a bag net installed at the drainage gate of the pond. Taking advantage of the positive phototactic behavior of the species during the night, shrimps are attracted by a lamp to the drained water stream and collected at the bag net, until the pond is dry. At 60 days of cultivation, based on synchronization with the ecdysis period, the total capture in the ponds was conducted, and the total biomass harvested per pond was quantified (B). Characterization of the intestinal tract microbiota was based on the sampling performed during the harvest that included 200 shrimp were collected per test pond, 1 Control pond and 2 replicate ponds of the MOS condition; the weight (W) was recorded for each individual, and the digestive tracts were dissected under aseptic conditions for all individuals in the samples. The removed digestive tracts were divided into 3 groups per pond, homogenized for the extraction of DNA and immediately preserved at −40 °C. In our study, we chose to increase the number of individuals because this is commercial culture with a total of 1.7 x 10^6^ shrimps. Hence, pools included 200 shrimps to obtain a more comprehensive representation of the microbiota and its variations. The extracted digestive tracts were pooled and homogenized by pond, according to the observations described in previous publications; in which pooled samples and individual samples were similar in terms of microbiota profiles^70^. It is thus a common practice to study the gut microbiota in fish using pooled samples^70–73^.

### Statistical analysis

Specific weight gain was determined as WG = (W_F_ − W_I)_/t, where W_F_ represents the average final weight, W_I_ represents the average initial weight, and t represents the time in days of cultivation. The survival rate (S) of shrimp in each pond was determined by the following formula: S = 100 × (n_T_/N_0_), where S is the survival rate, n_T_ is the number of shrimp harvested at the time t, and N_0_ is the number of shrimp at the beginning of the experiment. The conversion of food to biomass ratio was calculated using the following equation: FCA = AT/B, where AT represents the total food supplied, and B represents the biomass harvested.

To compare the productivity indexes for each treatment, we used Welch’s t-test^74^.

The survival (S) per condition was studied via an analysis of contingency tables using Pearson’s *X*^2^ test^75^.

### DNA extraction

For the DNA extraction, 100 mg of each homogenized subsample was taken. The sample was incubated with 0.8 mg/ml lysozyme (Merck, Germany) and (0.8 mg/ml) lyticase (Sigma-Aldrich Corp, US) for 1 hour at 37 °C. The sample was later incubated with (0.1 mg/ml) proteinase K (Ambion/Life Technologies, CA, USA) for another hour at 37 °C. Next, the protocol for the PowerSoil ® DNA Isolation Kit from MoBio (Mo Bio Laboratories Inc., Qiagen, Carlsbad, CA, USA) was used according to the manufacturer’s instructions.

### PCR amplification

To analyze the composition of the microbiota, part of the hypervariable V2-V3 region of the 16S rRNA gene was amplified. The PCR was performed using the primers 341F (5′ GCCTACGGGAGGCAGCAG 3′) and 518R (5′ CGTATTACCGCGGCTGCTGG 3′)^67^. The DNA concentration was determined using the Qubit dsDNA BR Assay Kit (Life Technologies, Grand Island, NY, US). Each reaction took place in a 30-μl solution consisting of 1 μl ≈ 1 ng of DNA, 18.5 μl of sterile, deionized deoxyribonuclease-free water, 6 µl of 5X buffer, 1.5 mM of MgCl_2_, 0.5 mM dNTPs, 0.84 μM each of forward and reverse primers and 0.5U of GoTaq (Promega, US). The PCR was performed in a Swift MiniPro thermocycler (Esco, China). The PCR program included an initial denaturation at 94 °C for 10 min, followed by 30 cycles of denaturation at 94 °C for 1 min, annealing at 53 °C for 1 min and 72 °C for 1 min for extension, with a final incubation of 72 °C for 10 min. The resulting amplicons were visualized using PAGE^76^ and then purified using a QIAquick PCR Purification kit (Qiagen, Crawley, UK) according to the manufacturer’s protocol.

### High-performance mass sequencing

A Qubit Fluorimeter (Invitrogen, CA, USA) was used to quantify the purified PCR products. The amplicons were then evaluated for fragment concentration using Ion Library Quantitation Kit (Life Technologies, CA, USA). The concentration was adjusted to 26 pM. The amplicons were attached to ion sphere particles (ISPs) using the Ion PGM kit Template OT2 400 (Life Technologies, CA, USA) according to the manufacturer’s protocol. Multiplexed sequencing was conducted using the 318 chip (Life Technologies, CA, USA) on the Ion Torrent Personal Genome platform (Life Technologies, CA, USA). Sequences were sorted by sample and filtered within the PGM software to remove low-quality reads. Finally, the data for each sample were exported to an individual Fastq file.

### Data processing of the high-performance mass sequencing results

The quality of the Fastq sequences obtained was analyzed by using prescreening in the program FastQC from Babraham Bioinformatics^77^. Then, the parameters retrieved (p: minimum number of consecutive high-quality base calls to retain read; q: last quality score considered low quality; r: maximum number of consecutive low-quality base calls allowed before truncating a read; and n: maximum number of ambiguous (N) characters) were used to filter our reads using the UPARSE pipeline according to the recommendations of Bokulich *et al*.^78^ and Edgard^79^. The sequences were merged in a single FASTQ file using the script Split_libraries_fastq.py from QIIME (version 1.9.1-20150604)^79^. Then, the UPARSE pipeline was used to perform chimera checking and OTU clustering, using the default parameters of 97% identity with the biological sequence as described Edgar 2013^79^. Taxonomic designations by RDP^80^ and sequence alignment with Pynast^81^, with a minimum threshold length of 170 bp, were performed in the QIIME pipeline^82^, using the 123 SILVA open source version as a reference^83^ with a threshold of 0.55 assigned by QIIME. The remaining sequences were considered unclassified, and the archaeal sequences were removed from the table and located in a separated table (see Suppl. Data). QIIME was also used to calculate a phylogenetic tree using the default parameters^84^ and 123 Silva as a reference database. Alpha diversity metric was obtained in the rarefied OTU tables. Beta diversity was evaluated using the Bray-Curtis method^85^ and is presented using principal component analysis (PCoA) based on the QIIME pipeline. The identification of OTUs with differential abundance between treatments was performed using MetagenomeSeq’s fitZIG method^86^, in QIIME. The comparison of the composition of the microbiota between treatments with the ANOSIM test^87^ were the last steps in the QIIME pipeline. To compare the microbiota associated with the treatments in terms of both statistical significance and biological relevance, we used the Linear Discriminant Analysis (LDA) Effect Size (LEfSe)^88^.

## Supplementary information

Supplementary material.
