# Supplementary material for: Effect of mannan oligosaccharides on the microbiota and productivity parameters of Litopenaeus vannamei shrimp under intensive cultivation in Ecuador
Source: Sci Rep. 2020 Feb 17;10:2719. doi: 10.1038/s41598-020-59587-y (PMC7026423; doi:10.1038/s41598-020-59587-y)
Supplement: Supplementary file 1 — Supplementary material. [file 41598_2020_59587_MOESM1_ESM.docx]

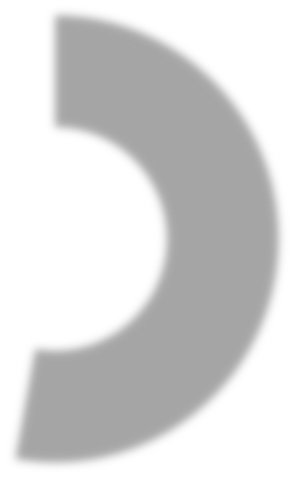

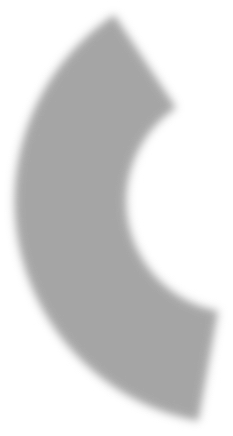

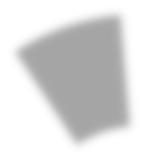

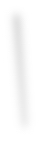

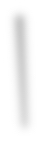

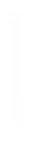

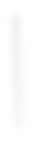

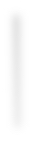

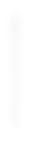

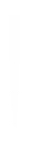

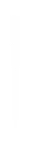

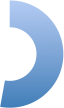

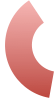

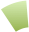

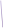

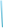

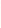

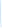

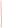

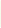

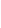

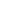

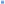

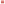

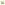

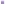

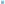

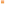

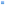

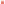

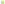

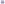

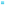

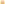

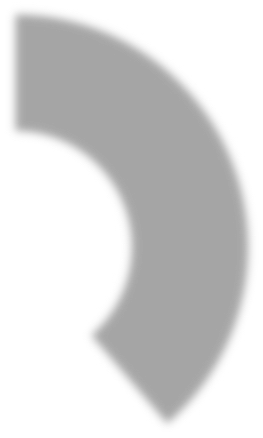

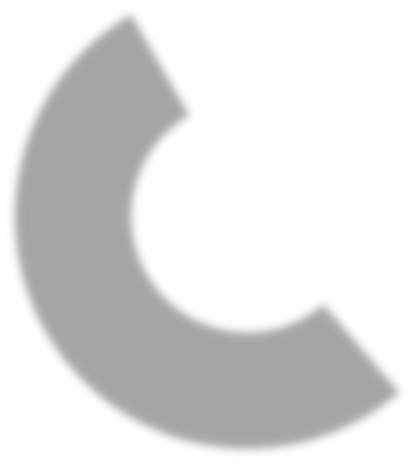

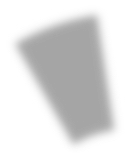

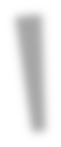

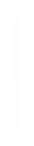

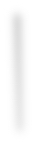

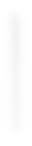

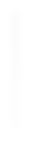

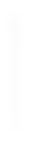

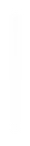

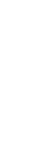

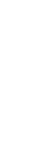

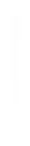

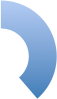

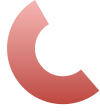

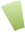

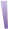

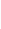

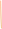

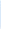

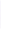

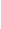

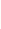

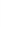

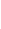

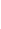

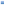

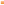

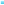


**Effect of mannan oligosaccharides on the microbiota and**

**productivity parameters of *Litopenaeus vannamei* shrimp under intensive cultivation in Ecuador**

Orestes Gainza Roche**^1^**, *****Jaime Romero**^2^**.

^1^Departamento de Acuicultura, Universidad Católica del Norte, ogainzar@gmail.com Facultad de Ciencias

del Mar. Coquimbo, Chile.

^2^INTA-UCH jromero@inta.uchile.cl Laboratorio de Biotecnología, Instituto de Nutrición y Tecnología de los

Alimentos (INTA), Universidad de Chile Avda. El Líbano 5524, Santiago, Chile.

*Correspondence and request for materials should be addressed to J. R. (email: jromero@inta.uchile.cl)

**Supplementary Material**

**Sample MOS 0.5% Control Total Mean**

**Number of SEQ** 727,590 575,902 1,303,492 144,832

**Phred Quality Score** 30(99.9%) 29(99.9%) 29(99.9%)

**High Quality SEQ** 155-179bp 149-179bp 149-179bp

**Quality filtering and Length trimming** 170bp 170bp 170bp

**# of SEQ post Quality and Chimeras Filtering** 265,228 239,397 504,625 252,312

**# SEQ post *Archaea* Filtering** 240,299 230,340 470,639 235,319

Supplementary Table 1. Characteristics of the sequences obtained.

**Control MOS**

Crenarchaeota Crenarchaeota

Thaumarchaeota Thaumarchaeota

Euryarchaeota Euryarchaeota

Diapherotrites Diapherotrites

Marine Hydrothermal Vent Marine Hydrothermal Vent

Group 1(MHVG-1) Group 1(MHVG-1)

Nanoarchaeota Nanoarchaeota

Aigarchaeota Aigarchaeota

Parvarchaeota Parvarchaeota

Woesearchaeota (DHVEG-6) Woesearchaeota (DHVEG-6)

Miscel aneous Crenarchaeotic Miscel aneous Crenarchaeotic

Group Group

Nanohaloarchaeota Nanohaloarchaeota

Marine Hydrothermal Vent Marine Hydrothermal Vent

Group(MHVG) Group(MHVG)

Supplementary Figure 1. Comparison of relative abundance of *Archaea* at the phylum level


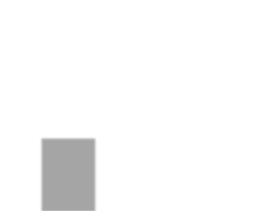

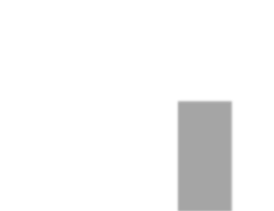

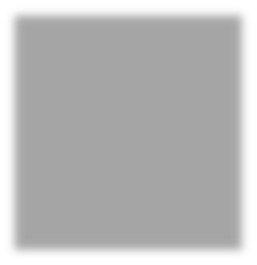

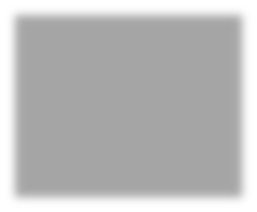

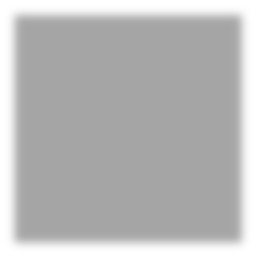

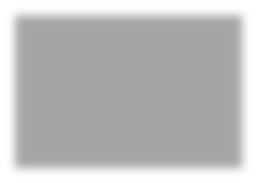

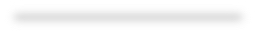

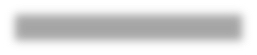

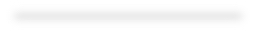

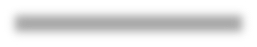

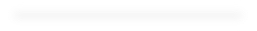

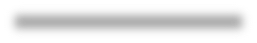

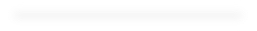

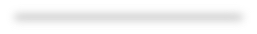

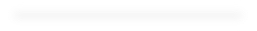

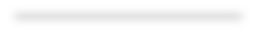

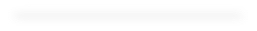

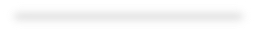

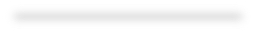

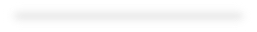

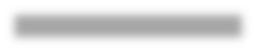

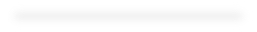

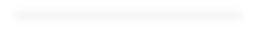

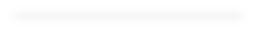

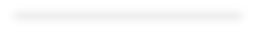

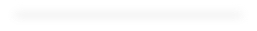

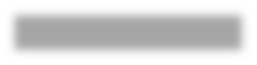

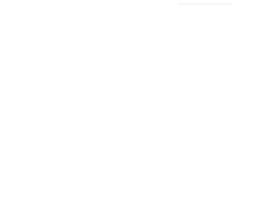
1

0.9

0.8

0.7

0.6

0.5

0.4

0.3

0.2

pSL12 Other

Candidatus Micrarchaeum

uncultured archaeon APA7-17cm

uncultured marine microorganism

HF130_10-06-02

uncultured sediment archaeon

Marine Group I Other

korarchaeote uncultured

korarchaeote;D_5__uncultured

korarchaeote

Pyrococcus

Halomicrobium

Haloferax

0.1

Pyrodictium

0 Methanosphaera

Control Mos

Supplementary Figure 2. Comparison of relative abundance of *Archaea* at the genera

level.
